# The different roles of innate immune receptors in inflammation and carcinogenesis between races

**DOI:** 10.1186/s12199-017-0678-8

**Published:** 2017-10-11

**Authors:** Natsu Yamaguchi, Yoshimi Suzuki, M. H. Mahbub, Hidekazu Takahashi, Ryosuke Hase, Yasutaka Ishimaru, Hiroshi Sunagawa, Rie Watanabe, Yoshinobu Eishi, Tsuyoshi Tanabe

**Affiliations:** 1 Department of Public Health and Preventive Medicine, Graduate School of Medicine, Yamaguchi University, 1-1-1 Minami-Kogushi, Ube, Yamaguchi 755-8505 Japan; 2 Department of Human Pathology, Graduate School and Faculty of Medicine, Tokyo Medical and Dental University, Tokyo, Japan

**Keywords:** TLR, NOD, Crohn’s disease, Sarcoidosis

## Abstract

Innate immune factors exert widespread effects on cytokine secretion, cell survival, autophagy, and apoptosis. Nucleotide-binding and oligomerization domain-like receptors (NLRs) are members of the innate immune system in the cytosol that sense pathogens, endogenous danger molecules such as uric acid, and pollutants. Nucleotide-binding oligomerization domain-containing protein 1 and 2 (NOD1 and NOD2) are components of NLR family, and ligands of these factors are γ-d-glutamyl-meso-diaminopimelic acid (iE-DAP) and muramyl dipeptide (MDP), respectively. Upon recognition of ligands, NOD1 and NOD2 induce the production of inflammatory cytokines and transcription factors including interleukin-6 (IL-6) and nuclear factor-κB (NF-κB). We examined the function of NOD1 and NOD2 in innate immunity, with a focus on their differing roles in disease pathogenesis between Japanese and Caucasian populations. Susceptibility to several immune-related diseases, including Crohn’s disease, colorectal and breast cancers, and graft-versus-host-disease (GVHD) showed a correlation with genetic variants of NOD2 in Caucasian, but not in Japanese, populations. This difference may be primarily due to the fact that three major NOD2 SNPs (R702W, G908R, L1007insC) prevalent in Caucasians are rare or absent in Japanese populations. Because NLR has diverse effects on immune function, it is possible that many as yet uncharacterized immune-related diseases will also show different susceptibilities between races due to the different ratio of genetic variants in innate immune genes.

## Background

**Fig. 1:**
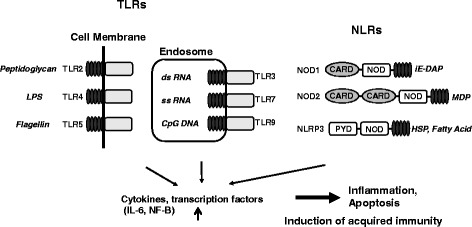
Innate immune receptors, NLRs and TLRs. The main members of the innate immune system are NLRs (membrane binding) and TLRs (in the cytosol). NLRs and TLRs sense intracellular and extracellular PAMPs, respectively. The subsequent activation of inflammatory cytokines including IL-6 and NF-κB results in the inflammation, apoptosis, and autophagy. These pathways are involved in the induction of acquired immunity

Two types of immune mechanisms, innate system and adaptive system, are used to eliminate the invading pathogens, endogenous danger molecules, and pollutants. As a first step of immune system, innate immune responses are mediated by a set of non-clonal, germline-encoded pattern-recognition receptors (PRRs) that sense conserved pathogen-associated molecular patterns (PAMPs) in pathogens and danger-associated molecular patterns (DAMPs). PAMPs include flagellin, single RNA, unmethylated CpG DNA, and lipopolysaccharides. DAMPs consist of endogenous factors such as fatty acids, heat shock proteins (HSP), and uric acid. Recent studies revealed that PRPs also recognize pollutants including asbestos [1] and PM2.5 [2]. Various PRRs exist in the extracellular space, integrated in cellular membranes, and in the cytosol. The toll-like receptors (TLRs) were first identified as genes that determine body axis. They represent a class of membrane-bound PRRs that respond to PAMPs and DAMPs at the cell surface and within endosomes [3] (Fig. 1). The nucleotide-binding oligomerization domain (NOD)-like receptor (NLR) family consists of cytoplasmic PRRs that play a pivotal role in sensing PAMPs and DAMPs in the cytosol [4]. The NLR family consists of more than 20 cytosolic proteins that are characterized by the presence of a conserved tripartite domain structure: C-terminal leucine-rich repeats (LRRs) that are involved in sensing of ligands, a central NOD domain, and an N-terminal effector-binding domain, such as the Pyrin domain (PYD) and the caspase recruitment domain (CARD) [4].

### NOD1 and NOD2

**Fig. 2:**
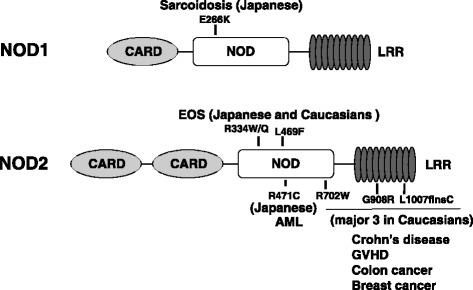
Mutations in NLR that cause immune-related diseases. NOD1 variant E266K associated with sarcoidosis was found in Japanese population. Three mutations in NOD domain of NOD2 (R334W, R334Q, and L469F) exist both in Blau syndrome in Caucasians and EOS in Japanese cohort. NOD2 SNPs near or within the LRRs of Nod2 (R702W, G908R, L1007insC) are associated with the development of CD in Caucasians that does not exist in Japanese cohort

NOD1 and NOD2 are members of the NLR family with an N-terminal CARD domain (Fig. 2). In early studies, NOD1 and NOD2 were found to induce nuclear factor-κB (NF-κB) activation when overexpressed in mammalian cells and to eliminate pathogens independently of TLRs [5]. The ligand for NOD1 is γ-d-glutamyl-meso-diaminopimelic acid (iE-DAP) found in many gram-negative and certain gram-positive bacteria [6]. As ligand, NOD2 recognizes muramyl dipeptide (MDP), a component of peptidoglycan that is present in both gram-positive and gram-negative bacteria [7]. Nod1 and Nod2 undergo conformational changes upon recognition of ligands, resulting in self-oligomerization via the central NOD domain and binding to receptor-interacting protein (RIP)-like interacting caspase-like apoptosis regulatory protein kinase (RICK, RIP2), a serine threonine kinase that leads to NF-κB activation [8]. The tissue distribution patterns of NOD1 and NOD2 are quite different. Expression pattern of Nod1 is ubiquitous in various tissues and Nod2 is restrictedly expressed in the Paneth cells of the small intestine and monocytes [4]. In vivo studies have revealed that NOD1 ligands simulate chemokine production and recruitment of neutrophils [9] and contribute to adaptive immune responses.

### Crohn’s disease

Crohn’s disease (CD) is characterized by recurring inflammation in the gastrointestinal tract. We reported the association between genetic polymorphisms of NOD2 and susceptibility to CD in Caucasians [10] (Fig. 2). The genetic variants of NOD2 found in about 10% of CD patients, including three common mutations that involve amino acid residues in NOD domain (R702W) or within LRRs (G908R and L1007insC) of Nod2, showed association with the development of CD. Ethnic differences in the genetic susceptibility to CD have been reported between Caucasian and Asian populations. Several studies revealed that none of the three common NOD2 variants that have been associated with CD in Caucasians are present in Japanese [11], Chinese [12], and Korean [13] CD patients. Also, a systemic genomic screening of the entire coding region of NOD2 in a Japanese CD population showed no common genetic variants [14]. These results apparently indicate that the NOD2 gene is not a major contributor to CD susceptibility in Asian population. Our functional analysis showed that CD-associated mutations lead to the reduction (R702W, G908R) or loss (L1007insC) of NF-κB activation upon MDP stimulation, compared with the wild-type NOD2 [4].

The plausible mechanism to explain the association between mutants of NOD2 and susceptibility to CD was estimated by constructing NOD2-deficient mice [15]. The mice showed that the NOD2 protein is a key regulator of immunity within the intestine. These animals failed to recognize bacterial muramyl dipeptide and were susceptible to bacterial infections through the oral route but not via the peritoneal or intravenous route. Further, it turned out that NOD2 was necessary to express defensins, a subgroup of intestinal anti-microbial peptides. The estimated hypothesis was that the dysfunction of NOD2 may lead to facilitated entry of bacteria into epithelial cells through defective regulation of defensin expression, leading to an abnormal inflammatory response to uncleared bacteria.

### Blau syndrome (BS) and early-onset sarcoidosis (EOS)

BS is a rare autosomal dominant disease characterized by early-onset granulomatous inflammation including uveitis, arthritis, and dermatitis with camptodactyly [16]. Because BS susceptibility locus has been identified at 16p12 to which coincides with that of NOD2, genetic screening of NOD2 were performed in families with BS [17]. BS families shared three missense mutations (R334Q, L469F, and R334W) in NOD2 (Fig. 2). We analyzed the function of these mutations and found that all of them augmented NOD2 basal activity, even in the absence of the ligand MDP [18], and further enhanced NOD2 activity by addition of MDP. Thus, the genetic variants in NOD2 associated with BS function as hyper-responsive mutations, which is consistent with the dominant mode of inheritance of the granulomatous disease.

EOS is a systemic granulomatous syndrome sharing the distinct triad of skin, joint, and eye inflammation with BS and is progressive and causes severe complications, such as destructive arthropathy and blindness. Systemic analysis of NOD2 genes of the EOS cases in Japanese population also showed the same mutations as in BS [19]. Thus, EOS shares a common genetic etiology of NOD2 with BS.

### Sarcoidosis

Sarcoidosis, a chronic systemic granulomatous disease of unknown cause, may result from the exposure of genetically susceptible subjects to a specific environmental agent(s). The only bacterium to be isolated from sarcoid lesions to date is *Propionibacterium acnes* (*P*. *acnes*) [20]. We found that both NOD1 and NOD2 proteins recognize intracellular *P*. *acnes*. Systematic search for NOD1 gene polymorphisms in Japanese sarcoidosis patients identified significant elevation of G796A in sarcoidosis patients (Fig. 2). Genetic screening of the NOD2 gene in sarcoidosis revealed no common mutations in Japanese and Caucasian subjects [21, 22]. Our functional analysis revealed that the NOD1 G796A was associated with lower expression in protein level leading to reduced NF-κB activation in response to intracellular *P*. *acnes* [23]*.* These results indicate that impaired recognition of intracellular *P*. *acnes* through NOD1 variant causes the susceptibility to sarcoidosis in the Japanese population.

### Malignant diseases

Mutations in the *NOD2* gene associated with Crohn’s disease have also been associated with an increased risk for the development of different types of cancer (*Helicobacter pylori*-induced MALT lymphoma, colonic adenocarcinoma, and breast and lung cancer) in Caucasians [24–26]. In Japanese populations, we revealed that a NOD2 SNP in the NOD domain (R471C) has been associated with acute myeloid leukemia (AML) but not with acute lymphoblastic leukemia [27] (Fig. 2). This difference may be explained by the results of recent studies that revealed an important role of NOD2 in the differentiation of bone marrow CD34^+^ hematopoietic cells, by mediating the induction of cytokines indispensable for cell differentiation toward the myeloid lineage [28]. It is possible that the NOD2 variant causes abnormal cell differentiation by altering cytokine production, thus leading to the elevated incidence of AML. There are several possible mechanisms that may account for the contribution of NOD2 to the risk of malignant diseases. Impairment of the innate immune system by NOD2 variants may induce chronic activation of alternate recognition receptors, such as the TLR, which can also lead to NF-κB activation and pro-inflammatory cytokine release, possibly provoking the inflammation-dysplasia-carcinoma sequence [29]. In addition to its role in the non-specific innate immune system, NOD2 may generate signals for the adaptive immune response, through cytokine release, leading to the activation of dendritic cells and the promotion of T cell differentiation. NOD2 variants may impair the adaptive immune system in evoking anti-tumor immunity [30].

### Graft-versus-host disease (GVHD)

Several recent studies in Caucasian patients have described a significant correlation between the severity of acute GVHD and the presence of three NOD2 SNPs: R702W, G908R, and L1007insC. Holler et al. reported an increased incidence and severity of acute GVHD associated with the presence of NOD2 SNPs in two separate patient cohorts [31, 32]. In contrast, Granell et al. demonstrated that in a T cell-depleted setting, these SNPs had no effect on acute GVHD [33]. In our analysis in a severe GVHD Japanese patient group, no genetic alteration of NOD2, including the three major SNPs seen in Caucasians, were found [26]. Previous studies have shown that Japanese and Swedish patient populations have a lower probability of acute GVHD [34]. No dysfunctional NOD2 variants were found in the severe acute GVHD group that may explain the differences in the occurrence of acute GVHD among different ethnic populations.

## Conclusions

We discussed the physiology of NLR-related diseases and ethnic differences between Caucasians and Japanese. The three mutations of NOD2 cause increased susceptibility to Crohn’s disease and several other malignant diseases, including colorectal and breast cancers in Caucasians, but the Japanese population lacks the NOD2 mutations. Because NLR exerts widespread effects on cytokine secretion, cell survival, autophagy, and apoptosis, it is possible that many as yet uncharacterized diseases will also show differences in susceptibility between races.

## Abbreviations

AML: Acute myeloid leukemia
BS: Blau syndrome
CARD: Caspase recruitment domain
CD: Crohn’s disease
DAMPs: Danger-associated molecular patterns
EOS: Early-onset sarcoidosis
GVHD: Graft-versus-host-disease
iE-DAP: γ-d-glutamyl-meso-diaminopimelic acid
IL-6: Interleukin-6
MDP: Muramyl dipeptide
NF-κB: Nuclear factor-κB
NLRs: Nucleotide-binding and oligomerization domain-like receptors
NOD1 and NOD2: Nucleotide-binding oligomerization domain-containing protein 1 and 2
*P*. *acnes*: *Propionibacterium acnes*
PAMPs: Pathogen-associated molecular patterns
PYD: Pyrin domain
RICK: Receptor-interacting protein-like interacting caspase-like apoptosis regulatory protein kinase
RIP: Receptor-interacting protein
TLRs: Toll-like receptors
